# Non-Native *Eragrostis curvula* Impacts Diversity of Pastures in South-Eastern Australia Even When Native *Themeda triandra* Remains Co-Dominant

**DOI:** 10.3390/plants10030596

**Published:** 2021-03-22

**Authors:** Corinne Schlierenzauer, Anita C. Risch, Martin Schütz, Jennifer Firn

**Affiliations:** 1 Faculty of Science and the Centre for the Environment, Queensland University of Technology (QUT), 2 George Street, Brisbane, QLD 4001, Australia; jennifer.firn@qut.edu.au; 2 Swiss Federal Institute for Forest, Snow and Landscape Research (WSL), Zuercherstrasse 111, CH 8903 Birmensdorf, Switzerland; anita.risch@wsl.ch (A.C.R.); martin.schuetz@wsl.ch (M.S.)

**Keywords:** African lovegrass, endangered grassy woodlands, Kangaroo grass, native and exotic plants, native herbivorous marsupials, non-native herbivorous mammals, plant invasion, plant-herbivore interactions, species richness, Shannon diversity

## Abstract

Lowland grassy woodlands in Australia’s south-east face reductions in native plant diversity because of invasion by non-native plants. We compared the relative abundance and diversity of plant species among sites dominated by the native Kangaroo grass (KG) *Themeda triandra* with sites co-dominated by the non-native African lovegrass (ALG) *Eragrostis curvula* and KG. We found significant differences in plant species composition depending on the dominant species. Furthermore, our results revealed differences in several diversity parameters such as a lower species richness and forb diversity on sites co-dominated by ALG and KG. This was the case despite the functional similarity of both ALG and KG—both C_4_ perennial tussock grasses of a similar height. Therefore, our results highlight the critical function of the native KG in maintaining and enhancing the target plant species composition and diversity within these grassy woodlands. Herbivore grazing potentially impacts on the abundance of the dominant grass and forb species in various ways, but its impact likely differs depending on their evolutionary origin. Therefore, disentangling the role of individual herbivore groups (native-, non-native mammals, and invertebrates) on the plant community composition of the lowland grassy woodlands is essential to find appropriate grazing regimes for ALG management in these ecosystems.

## 1. Introduction

The accidental or intentional introduction of non-native plants alters ecosystems by changing nutrient cycling [1,2], modifying disturbance regimes [3,4], and displacing native plant species [5,6]. Australian ecosystems, such as lowland grassy woodlands, are particularly threatened by non-native species. These grassy woodlands are usually comprised of an open tree canopy and ground-cover dominated by grasses and herbs. Extensive habitat clearing and degradation resulted in their drastic decline, and nowadays they are listed as an endangered ecological community [7,8].

*Eragrostis curvula* (Schrad.) Nees (African lovegrass, hereafter ALG), a C_4_ perennial grass originating from subtropical southern and eastern Africa [9], was introduced from the early 1900s to the 1980s [10]. Today, it can be found in every state and territory of Australia, but it is widespread and abundant mainly in the southern regions and parts of Queensland [9,11]. In contrast to initial expectations that ALG would improve pastures and stabilize soils, ALG turned out to have a low palatability to livestock, especially when its tissue matures [12,13]. Grazer avoidance not only enhances the abundance of ALG, but also has severe economic and social impacts in agricultural regions by reducing overall farm productivity [14]. Further, ALG has been shown to change plant species composition by forming dense tussocks and dominating the ground layer, thus reducing plant diversity [15,16].

Finding a sustainable and an effective long-term management strategy for weeds such as ALG is essential, but at the current stage, the eradication of ALG is unrealistic, hence controlling its further spread is the only option [17]. Landholders use different approaches to achieve this: Spot spraying, slashing, roller wiping, or the use of natural or chemical fertilizers. Despite the partial success of some of these strategies, they also come with a variety of disadvantages. For instance, a previous study showed that spot spraying can control ALG, but it seems effective only at early stages of invasion [16]. Although fertilizer application increased the nutrient content and the palatability of ALG and reduced its abundance [18], it is not sustainable in the long-term, as repeated fertilization increases the nutrient level of these sites. This has negative consequences for the native plant communities, as these sites are naturally low in nutrients [19,20].

A promising strategy to shift the grassland towards a more diverse plant community may be through grazing management including modifying the timing, intensity, and spatial patterns of grazing [21]. There is some evidence that mammalian herbivores of different evolutionary backgrounds could help in reducing non-native grass cover, as it has been shown that wildlife grazing increased native species richness, whereas livestock grazing increased non-native species richness in semi-arid rangelands of eastern Australia. These opposing effects of mammalian herbivores of different evolutionary backgrounds was stronger in low productivity systems [22].

Overall, pasture management strategies that help to reduce non-native grass cover and enhance biodiversity but do not deter long-term agricultural yield remain equivocal [23]. Before such pasture management strategies can be developed and implemented, we need to better understand how ALG alters the critically endangered lowland grassy woodlands under current conditions.

Therefore, the goal of this study is to identify differences in plant species composition and richness among sites dominated by native Kangaroo grass (i.e., *Themeda triandra* Forssk, hereafter KG) and sites co-dominated by KG and ALG. We selected six farms for our study, and on each farm, we established two sampling sites, one dominated by KG (hereafter referred to as KG sites) and the other co-dominated by ALG and KG (hereafter ALG+KG sites). All farms are grazed by livestock, mainly sheep and cattle. Other herbivores present are native marsupials such as eastern grey kangaroos (*Macropus giganteus* (Shaw, 1790)), red-necked wallabies (*Macropod rufogriseus* (Desmarest, 1817)), swamp wallabies (*Wallabia bicolor* (Desmarest, 1804)), and common wombats (*Vombatus ursinus* (Shaw, 1800)) as well as invertebrates (grasshoppers, cicadas, aphids, etc.). Data was collected in autumn (May) and spring (November) 2020 as these grassy woodlands have two distinct seasons due to the local climate regime.

Specifically, we addressed the following research questions:

- How do ALG+KG sites differ in plant species richness and diversity (overall and within functional groups) compared to KG sites?
- What is the relationship between native and non-native plant species richness and diversity at each of the two site types?
- How does the cover of functionally different plant groups and lifeforms vary among KG and ALG+KG sites?
- Are there compositional differences among KG and ALG+KG plant communities and which species explain potential differences?

## 2. Results

Overall, we found 92 plant species of which 45 were native and 37 were non-native. Ten species were only identified to the genus and therefore not classified with regard to their origin. 56 of the species found were forbs (49 non-legumes, 7 legumes) and 33 were graminoids (24 grasses, five sedges, four rushes). The remaining species were trees or ferns. On average, we recorded almost 16 species per 1 × 1 m^2^ plot. The mean ALG and KG cover for both site types and seasons is given in Table 1.

### 2.1. How Do ALG+KG Sites Differ in Plant Species Richness and Diversity (overall and within Functional Groups) Compared to KG Sites?

**Figure 1:**
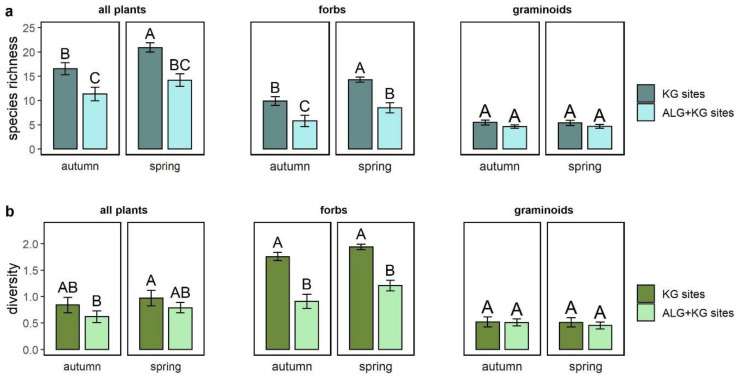
(**a**) Mean species richness and (**b**) Shannon diversity per plot for all plants (left), only forbs (middle), and only graminoids (right) on KG (darker colour) versus ALG+KG (lighter colour) sites. Different capital letters indicate significant differences (*p* < 0.05) among site types and sampling season. Error bars represent the standard error of the mean.

Total plant species richness was higher on KG sites compared to ALG+KG sites in both seasons (Figure 1a). Specifically, mean species richness on ALG+KG sites was 35% and 31% lower compared to KG sites in spring and autumn, respectively. These differences were mainly caused by a higher number of forbs on KG sites, whereas there was little difference in graminoid species richness between site types. In addition, we found on average of about four species more in spring compared to autumn on KG sites, whereas species richness did not differ on ALG+KG sites between the two seasons.

Overall Shannon diversity did not differ among site types (*p* = 0.14). However, we found evidence that forb diversity was significantly higher on KG sites, whereas no significant difference was found in the diversity of graminoids between the two site types (Figure 1b).

### 2.2. What Is the Relationship between Native and Non-Native Plant Species Richness and Diversity at Each of the Two Site Types?

**Figure 2:**
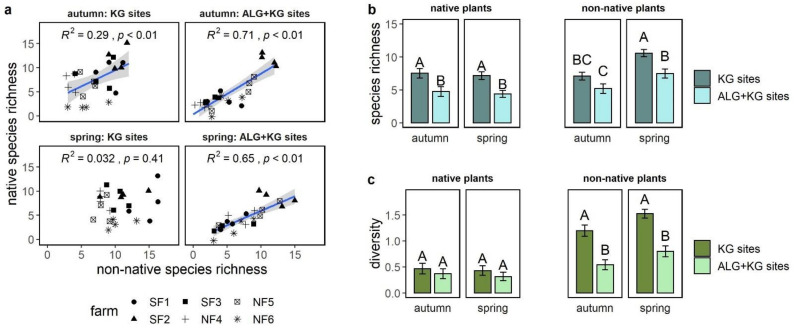
(**a**) Correlation among mean native and mean non-native species richness per plot for each site type and season, including Pearson correlation coefficient (R^2^) and *p*-value. (**b**) Direct comparison of mean native and mean non-native species richness, and (**c**) the same for Shannon diversity per plot for KG (darker colour), ALG+KG (lighter colour) sites in autumn and spring. Different capital letters indicate significant (*p* < 0.05) differences among the compared groups and error bars represent the standard error of the mean.

On plots with high native species richness, we also found high non-native species richness, except for the KG sites in spring (Figure 2a). This positive correlation was stronger on ALG+KG sites than KG sites. We found no such correlations for Shannon diversity (Supplementary Material: Figure S1).

In spring, the mean number of non-native species was about 50% higher than the mean number of native species in both site types. This was not the case in autumn, where a similar number of native and non-native species was found on ALG+KG sites and slightly more non-native plants on KG sites (Figure 2b). Comparing the site types, we found that the mean native species richness was around 38% (*p* < 0.05) higher on KG sites in both seasons; whereas, mean non-native species richness was 37% (*p* = 0.05) higher on KG sites in autumn and 30% (*p* < 0.05) higher in spring.

There was no difference among the site types and seasons for native Shannon diversity, whereas non-native Shannon diversity was higher on KG sites compared to ALG+KG (autumn: 120% higher and spring: 90% higher; Figure 2c). However, when we considered only native forbs, we found a higher Shannon diversity on KG sites compared to ALG+KG sites (spring: 102%, autumn: 148%). In concert with the positive correlation found between native and non-native species richness, we also found a similar result of higher non-native forb Shannon diversity on KG sites (60% higher in spring, 108% higher in autumn).

### 2.3. How Did the Cover of Functional Groups and Plants with Different Lifeforms Vary among KG and ALG+KG Sites?

**Figure 3:**
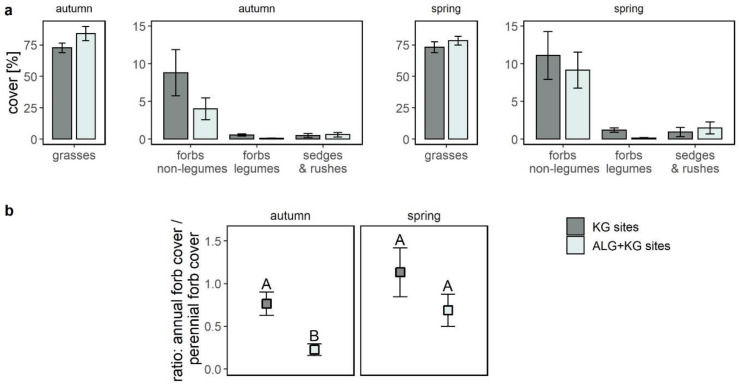
(**a**) Composition of functional groups for KG (darker colour) and ALG+KG (lighter colour) sites in autumn and spring with the mean cover per functional group for each plot including the standard error as error bars. (**b**) The mean ratio of annual forb cover to perennial forb cover per plot is given for each site type with the standard error as bars. Different capital letters indicate significant differences (*p*-value < 0.05) among site types.

Grass cover was similar at both site types (Figure 3a), with a mean cover between 72.7% ± 3.9% (KG sites spring) and 84.0% ± 5.7% (ALG+KG sites autumn). Sedges and rushes had a very low cover regardless of site type and season (Figure 3a). The mean cover of non-leguminous forbs was much higher (factor of 2.2) on KG sites in autumn and slightly higher (factor of 1.2) in spring compared to ALG+KG sites. In both seasons we found a higher cover of legumes on KG compared to ALG+KG sites, with a factor of 5.1 and factor of 7.4 in autumn and spring, respectively. The graminoids were dominated by perennial grasses, whereas the annual graminoid cover was very low on both site types in spring (ALG+KG: Mean 0.06% ± se 0.03% and KG: Mean 0.02% ± 0.01%) and zero in autumn. The forb cover was more balanced between perennial and annual lifeforms. Looking at the ratio of the annual to perennial forb cover (Figure 3b), there was a significantly (*p* < 0.05) lower ratio on ALG+KG sites in autumn, but no significant difference was found in spring (*p* = 0.27). No such ratio was calculated for the graminoids due to the absence of annual graminoid cover in autumn.

### 2.4. Are There Compositional Differences among KG and ALG+KG Plant Communities and Which Species Explain Potential Differences?

**Figure 4:**
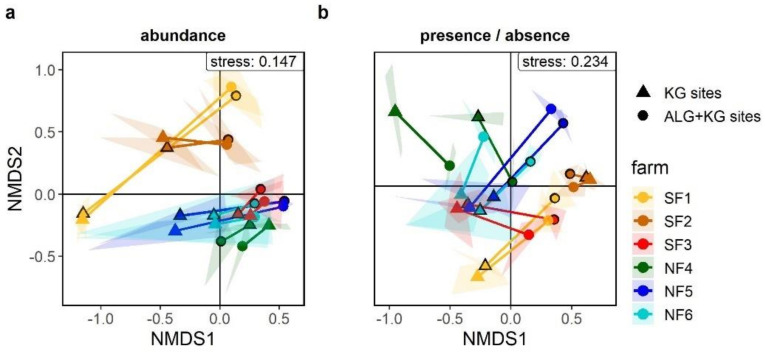
nMDS with input data based on (**a**) the abundance and (**b**) presence/absence of plant species using both spring and autumn data in each graph. Each farm is illustrated with a different colour, whereas each polygon represents a site, and the lines connect the centroids of the KG (triangle) and the corresponding ALG+KG (circle) site of the same farm. A black line around the centroid symbols represents the spring data, whereas its absence stands for the autumn data. The letters S and N in the farm name represent the southern group (S) and the northern group (N) of farms.

We performed non-metric multidimensional scaling (nMDS) and permutational MANOVA (PERMANOVA) to identify differences in plant species composition among KG and ALG+KG sites. We found significant differences in plant community composition between the two site types (site type: *p*-value < 0.01, *R*^2^ = 0.12, *F*-value = 29) and farms (farm: *p*-value < 0.01, *R*^2^ =0.35, *F*-value=18; Figure 4a). To ensure that the differences did not simply originate from variations in ALG and KG abundance between the two site types, we also analysed presence/absence species data. This revealed similar differences in species composition among site types (site type: *p*-value < 0.01, *R*^2^ = 0.09, *F*-value = 21) and farms (*p*-value < 0.01, *R*^2^ = 0.34, *F*-value = 17) as percent cover (Figure 4b).

Similarity percentages (SIMPER) analysis indicated a dissimilarity between the site types of approximately 58% for spring and autumn (Table 2). These differences in vegetation composition between KG and ALG+KG sites were mainly driven by KG and ALG, as both contributed to around 18% of the overall dissimilarity, whereas all the other species contributed to less than 5%. Looking at the SIMPER output for the species presence/absence data, we found a dissimilarity among the KG and ALG+KG sites of 68% for spring, and about 61% for autumn. None of the species was contributing more than 3.5% to the overall dissimilarity. Complete results of the SIMPER analyses for both abundance and presence/absence date (spring and autumn) can be found in Supplementary Tables S1–S4.

## 3. Discussion

Overall, we have found that sites co-dominated by ALG and KG have a lower plant species richness and diversity compared to sites dominated by native KG. The lower species richness on sites with a high ALG cover was mainly driven by a lower number of forb species. One reason for the lower forb species richness and diversity on ALG+KG sites may be the higher graminoid cover on these sites. This can lead to competition for ground-level light, space, and nutrients [24]. Therefore, species that need more light, such as some growth restricted forbs, may have difficulties in surviving. Annual species may not have the microsite and light conditions necessary to germinate, which is supported by their lower abundance on the ALG+KG sites. In addition, ALG can restrict the movement of livestock through pasture [15] and is considered to have a low palatability, particularly when mature [12,13]. Livestock may therefore prefer to graze in communities with a low ALG abundance, resulting in lower grazing intensity on patches with high ALG cover [15,25]. This can lead to a further increase in ALG cover, to higher understory light limitation and, in turn, to a boosted decline of plant diversity [26].

How much native versus non-native plant species contribute to overall plant species richness has been addressed in several studies, but no consistent patterns were found. Invasion of non-native species could have both additive effects on overall richness [27] or negative effects on native and thus overall richness [28]. The spatial scale of the study [29], environmental conditions, biogeographical context, and introductory histories of non-native plants [30] are important drivers of these seemingly conflicting results, referred to as the invasion paradox. We found a positive correlation between native and non-native species, i.e., an additive effect of invasion on overall species richness. Our results may be explained by the biotic acceptance hypothesis, indicating that preferred conditions for native species are also beneficial for non-native species [31]. Such positive relationships between native and non-native species richness on small spatial scales (<1 m^2^) are often found in dispersal and immigration-driven communities as well as disturbed sites such as roadsides or agricultural landscapes [29,32]. A stronger disturbance, e.g., due to ALG management practices on ALG+KG sites, may therefore explain the stronger positive correlation found on these sites compared to KG sites. Yet, despite this positive correlation, we were unable to find a significant relationship between native and non-native Shannon diversity. A reason for this might be the comparatively low Shannon diversity of native plant species due to the dominance of native KG on all sites. This is likely due to the high relative abundance of the native KG on all sites resulting in a low value when calculating Shannon diversity index.

Although ALG and KG are functionally similar grasses, both of them tussock-forming C_4_ perennials [33] with similar heights (KG: up to 1.5 m [34]; ALG up to 1.2 m [35]), they differ in the framework structure they provide in grassland communities. Our compositional analyses showed that they affect the number and abundance of subordinate plant species differently within the community. KG has been traditionally present in grassy woodlands of south-eastern Australia and offers niches for the establishment of eucalypt seedlings and a variety of smaller plants such as forbs [36]. The lower forb diversity on ALG+KG sites indicates that ALG may not be able to provide the same conditions regarding inter-tussock spaces and establishment possibilities as KG. Therefore, appropriate native perennial tussock grasses such as KG may be critical to restore ecological processes [37]. A suitable herbivore grazing management regime is key in maintaining and enhancing KG, as this grass needs some grazing, but if the grazing pressure is too high, the result is its decline [38].

In addition, inappropriate grazing management has been shown to enhance invasion, establishment, and spread of non-native plants via several processes. For example, trampling by ungulates can lead to disturbances of the ground impeding the establishment of some native species, whereas non-native plants may be better adapted to such conditions. Another process is that herbivores contribute to the transport of non-native plant seeds from one place to another [39]. Furthermore, non-native plants are often more tolerant to livestock grazing [40], whereas Australia’s native plants are relatively poorly adapted to it [41]. Many non-native plants have a lower palatability, thus most herbivores feed selectively on the more palatable native plants. This can reduce their fitness, while leaving their less palatable neighbours unaffected [39,42]. Especially, in combination with certain abiotic conditions, grazing-sensitive species are more likely to decline and may be removed from the regional species pool [43].

Furthermore, the type of herbivore grazing can alter the invasion rate as well. Parker et al. (2006) [44] showed that native herbivores had a strong suppressive effect on the relative abundance of non-native plants, whereas non-native herbivores enhanced their relative abundance. Yet, other studies revealed that non-native herbivores considerably helped to control non-native plant species [18,41,45], indicating that the role of native versus non-native herbivores in controlling non-native plant species remains rather unclear.

The type of herbivore may also explain differences in the relationship between annual to perennial forb cover on KG versus ALG+KG sites. Sheep, cattle, and some native mammals such as eastern grey kangaroos seem to selectively feed on annual plants if available [46]. Annual grasses were low in presence and percent cover at both site types, which can either be due to natural conditions such as the drought from 2017 to 2019 [47,48], or the collective pressure of drought and herbivores. Likewise, the lower ratio of annual to perennial forb cover on ALG+KG could be explained by the reduced availability of palatable perennial grasses. This increases grazing pressure on forbs, specifically annual forbs, as they are preferred by many herbivores (e.g., sheep or eastern grey kangaroos) [46].

Despite their seasonal preference for annual grasses and forbs, the bulk food of eastern grey kangaroos are perennial grasses [46,49,50]. Other native marsupials such as red-necked wallabies or common wombats prefer grasses as well [51,52]. This can likely play an important role in maintaining and enhancing forb species diversity within the lowland grassy woodlands, as these native herbivorous marsupials may reduce dominant grass cover and thereby indirectly benefit forbs.

Whether the differences in forb diversity among KG and ALG+KG sites can be related to grazing remains unclear, however, as different studies showed contrasting results. For instance, Travers et al. (2017) [53] found that livestock and rabbit grazing reduced forb occurrence in semi-arid woodlands of south-eastern Australia. Other studies such as Zimmer et al. (2010) [54] found in a temperate grassland in south-eastern Australia that grazing combined with resting periods resulted in an increase of native, perennial, and non-native annual forb abundance, but not species richness. Generally, the impact of grazing on forb diversity seems to be inconsistent and can strongly vary with local conditions such as precipitation or grazing history [55].

Additionally, the density of herbivores seems to be relevant for plant responses. For example, Mutze et al. (2016) [56] found that increasing rabbit densities resulted in an exponential reduction of native pasture cover in South Australia. Yet, how type and density of herbivores affect plant richness and diversity also depend on abiotic parameters such as soil moisture and fertility [57,58,59]. Thus, finding a well-balanced mixture and density of native and non-native herbivores grazing within the same system might provide a solution for reducing non-native plant cover while enhancing critical native plants. Once the role of different herbivore groups (marsupials, non-native mammals, and invertebrates) on plant community composition within the lowland grassy woodlands is understood, new management schemes for a sustainable agricultural use of these ecosystems may be developed.

## 4. Materials and Methods

### 4.1. Study Area

**Figure 5:**
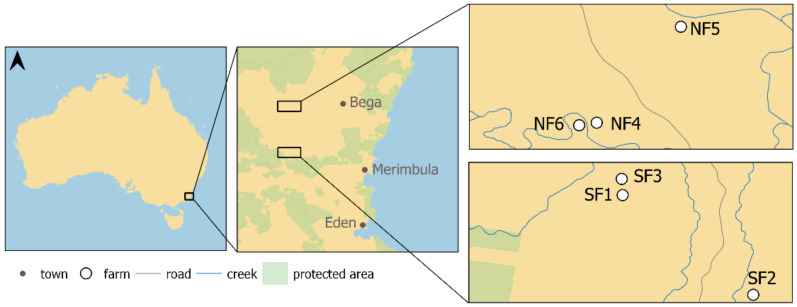
Map outlining the region of study including the location of farms with the experimental set-up. On each farm are two sites, one of them with a high ALG cover and the other dominated by native grasses (mainly KG). N = North, S = South, F = farm. The map was created and adapted from Open Street Map (OSM; provided by Geofabrik [62]) and the World Database of Protected Areas [63].

The Bega Valley Region is located in the southeast corner of New South Wales, Australia (Figure 5). Embedded between the Pacific Ocean and the Australian Alps, the lowland grassy woodlands are mostly located on granitic substrates and reach elevations of roughly 500 m above sea level. Typically, these grassy woodlands receive less precipitation compared to the more elevated areas that surround them, with a mean annual precipitation between 700–1100 mm [8].

The vegetation is dominated by an open tree canopy layer consisting of *Eucalyptus tereticornis* Sm, *Angophora floribunda* Sm. (Sweet) and a range of other eucalypt species. Sometimes shrub or small trees are also present, whereas grasses and forbs form the ground-cover. In areas without intensive agricultural history, this layer is dominated by perennial, tussock grasses such as KG, *Microlaena stipoides* R.Br (Weeping Grass), *Eragrostis leptostachya* Steud. (Paddock Lovegrass) and *Echinopogon ovatus* P.Beauv (Forest Hedgehog Grass). The remaining inter-tussock spaces are occupied by a diversity of growth-restricted grasses and herbaceous forbs [8,60].

Clearing, pasture sowing, fertilizer application, and livestock grazing resulted in a dramatic decrease in the extent of these natural woodlands, with less than five percent within conservation reserves and overall with only about 20% of their original extent in New South Wales still existing [61]. The remaining areas outside of reserves are threatened by altered fire frequencies, habitat clearing, livestock grazing, and especially by non-native plant invasion, particularly ALG. For this reason, the grassy woodlands are listed as an endangered ecological community in the NSW state legislation. Additionally, they are considered as critically endangered by the Commonwealth of Australia [8].

### 4.2. Experimental Design and Data Collection

For this study, six farms (Figure 5), and in each of them, two sites, were chosen, representing a paired design. One of the sites at each farm is dominated by native KG, the other one co-dominated by non-native ALG and KG. All twelve sites are within open pastures, with the presence of non-native and native herbivores. On each site, data was collected within four plots (each 1 × 1 m) in May and November 2020. All plant species found within a plot were recorded and their relative abundance was estimated.

### 4.3. Data Analysis

All data were analysed in R statistical computing version 4.0.2 [64]. We calculated species richness and Shannon diversity on plot level. Species richness represents the number of plant species per plot. Shannon diversity was calculated using the vegan R package (version 2.5-6) [65]. We estimated species richness and Shannon diversity overall for two functional groups (forbs, graminoids) and for native as well as non-native plant species separately. In addition, we determined the lifeform of each plant, whereby plants that can behave as annuals or perennials were included to the annual-lifeform category due to the very low number of such plant species. We calculated the cover per functional group and per lifeform for each plot and used the ratio of annual to perennial forbs.

Data were analysed using Linear Mixed Effect Models (LMM; lme4 package, version 1.1.23; [66]). For some cases, namely native species richness, native Shannon diversity, graminoid Shannon diversity, and the ratio of the annual to perennial forb cover, data were square root transformed to achieve normality of the residuals. However, data were back-transformed for all graphs. All other data were left untransformed. Fixed effects were time of sampling (autumn (May), spring (November)) and site type (ALG+KG, KG), whereas farm was added as random effect. We conducted post hoc comparisons of estimated marginal means and interaction effects between the sites types and month using the R package emmeans (version 1.5.2.1) [67]. In addition, we plotted native against non-native species richness as well as Shannon diversity and calculated Spearman correlation coefficient per season and site type.

Differences in plant species composition were analysed by multivariate statistical methods. To emphasize both the dominant and the medium abundant species, the relative abundance data were square root transformed [68]. We performed a PERMANOVA, using the pairwiseAdonis R package (version 0.0.1) [69] with Bray–Curtis similarity metric and 9999 permutations. To illustrate the multivariate patterns, we used nMDS on Bray–Curtis distance, and to identify dissimilarities, we conducted SIMPER analysis (vegan package [65]).

## Figures and Tables

**Table 1:** Mean ALG and KG cover per plot for both site types

	ALG Cover [%] (Mean ± SE)	KG Cover [%] (Mean ± SE)
KG sites (autumn)	3.3 ± 1.3	61.5 ± 4.7
KG sites (spring)	3.3 ± 1.3	64.0 ± 5.5
ALG+KG sites (autumn)	25.8 ± 6.4	46.6 ± 7.1
ALG+KG sites (spring)	28.8 ± 6.4	50.5 ± 8.4

Mean ± standard error (SE) of African lovegrass (ALG) and Kangaroo grass (KG) cover [%] per plot for both site types and seasons.

**Table 2:** Output of SIMPER showing the five most discriminating species (contribution to overall dissimilarity [%] whereby overall dissimilarity = 100%) among KG and ALG+KG sites. A * in front of the species name indicates its non-native origin. Overall dissimilarity for each season and model (abundance and presence/absence) is given in the last row. For full species lists, please see Supplementary Tables S1–S4.

	Autumn: Abundance	Spring: Abundance	Autumn: Presence/Absence	Spring: Presence/Absence
Top Five Species Contributing to Overall Dissimilarity	KG (18.6%)	* ALG (16.3%)	*Sporobolus elongatus* (3.5%)	* Facelis retusa (3.4%)
	* ALG (17.6%)	KG (15.3%)	*Glycine tabacina* (3.3%)	* Gamochaeta calviceps (3.0%)
	* Hypochaeris radicata (4.2%)	* Hypochaeris radicata (4.9%)	* Senecio madagascariensis (3.3%)	* Sisyrinchium rosulatum (2.9%)
	*Poa labillardierei* (4.1%)	* Plantago lanceolata (3.0%)	*Trifolium sp.* (3.2%)	*Glycine tabacina* (2.9%)
	* Cenchrus clandestinus (2.7%)	*Poa labillardierei* (3.0%)	* Hypochaeris radicata (3.2%)	* Senecio madagascariensis (2.8%)
Overall Dissimilarity	57.7%	58.2%	68.0%	61.8%

## Data Availability

The data presented in this study are openly available in EnviDat Repository at https://doi.org/10.16904/envidat.214 (accessed on 15 March 2021), reference number [70].

## Supplementary Materials

The following are available online at https://www.mdpi.com/2223-7747/10/3/596/s1, Figure S1: Correlation between native and non-native Shannon diversity, Figure S2: Correlation between mean native and mean non-native species richness per site, Table S1: Output of SIMPER analysis based on the abundance data in autumn, Table S2: Output of SIMPER analysis based on the abundance data in spring, Table S3: Output of SIMPER analysis based on the presence/absence data in autumn, Table S4: Output of SIMPER analysis based on the presence/absence data in spring.
