# Supplementary material for: Non-Native Eragrostis curvula Impacts Diversity of Pastures in South-Eastern Australia Even When Native Themeda triandra Remains Co-Dominant
Source: Plants (Basel). 2021 Mar 22;10(3):596. doi: 10.3390/plants10030596 (PMC8005164; doi:10.3390/plants10030596)
Supplement: Supplementary file 1 [file plants-10-00596-s001.pdf]

Supplementary Material

1. Correlation between native and non-native Shannon diversity

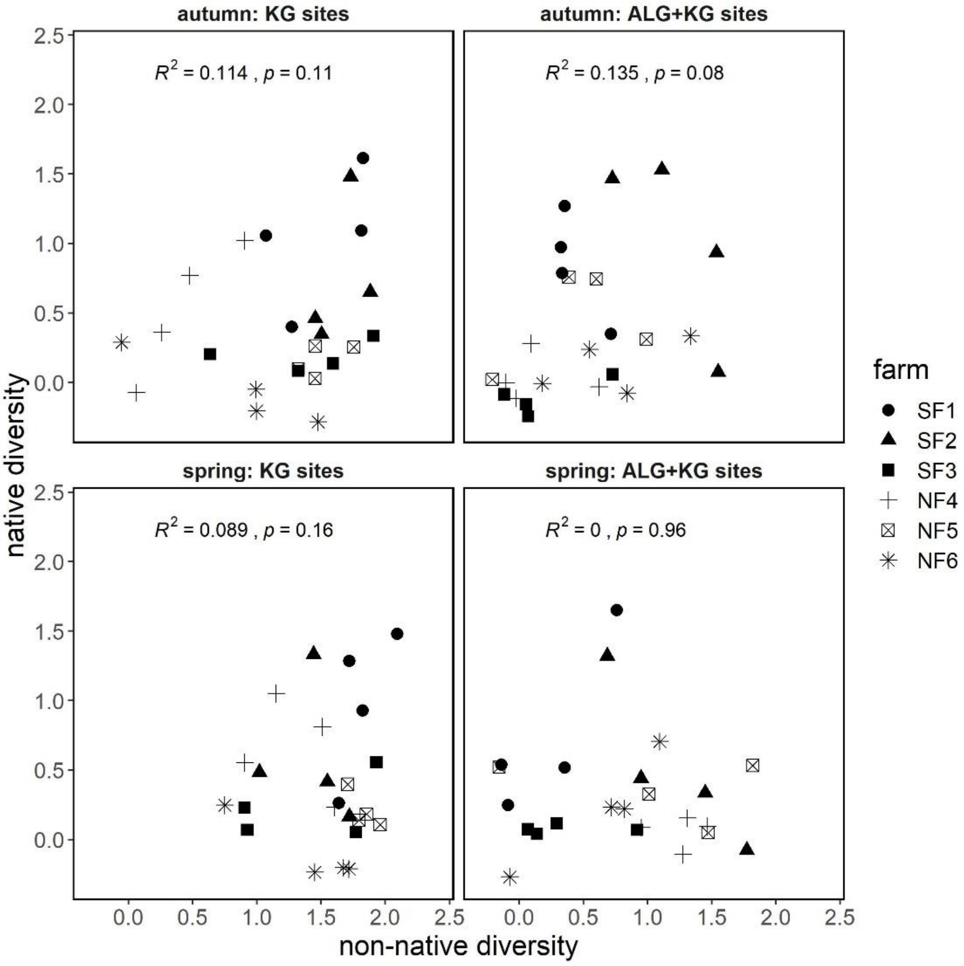

Figure S1: Correlation among native and non-native Shannon diversity per plot for each site type and season, including Pearson correlation coefficient (R^2) and p-value.

2. Correlation between native and non-native species richness

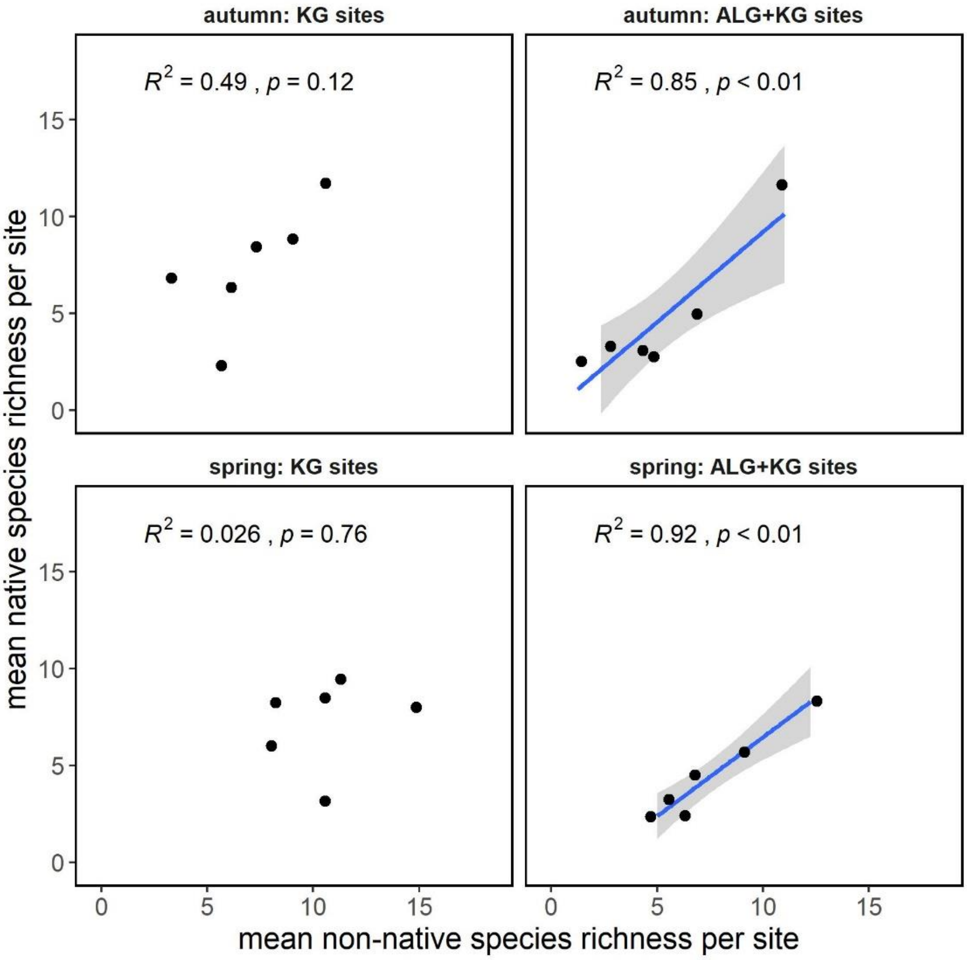

Figure S2: Correlation among mean native and mean non-native species richness per farm, separated for each site type and season, including Pearson correlation coefficient (R^2) and p-value.

3. SIMPER Output

Table S1. Output of SIMPER among KG and ALG+KG sites based on abundance data in autumn. Average: mean contribution to the overall dissimilarity, sd: standard deviation of the average dissimilarity, ava: average abundance per plot on ALG+KG sites, avb: average abundance per plot on KG sites, cumsum: ordered cumulative contribution.

species	average	sd	ava	avb	cumsum
Themeda_triandra	0.107129	0.09783	0.589004	0.767443	0.185702
Eragrostis_curvula	0.101558	0.095485	0.403701	0.124532	0.361746
Hypochaeris_radicata	0.024229	0.024681	0.085403	0.061683	0.403746
Poa_labillardierei	0.023365	0.048444	0.038975	0.050823	0.444248
Cenchrus_clandestinus	0.015312	0.027883	0.023287	0.047947	0.47079
Paspalum_dilatatum	0.015295	0.02162	0.046976	0.01927	0.497303
Microlaena_stipoides	0.015088	0.015925	0.030258	0.046606	0.523458
Paronychia_brasiliana	0.014975	0.022798	0.016979	0.058808	0.549416
Oxalis_perennans	0.014018	0.018268	0.015662	0.057472	0.573715
Plantago_lanceolata	0.013866	0.017979	0.028064	0.04438	0.597751
Sporobolus_elongatus	0.01368	0.015403	0.011398	0.046628	0.621465
Eragrostis_leptostachya	0.01233	0.021307	0.001318	0.051612	0.642838
Cirsium_vulgare	0.010929	0.021899	0.00527	0.044158	0.661783
Leontodon_saxatilis	0.010531	0.018021	0.016089	0.031743	0.680039
Carex_inversa	0.010325	0.01253	0.030711	0.0182	0.697936
Polycarpon_tetraphyllum	0.010102	0.024651	0.001318	0.044227	0.715448
Bothriochloa_macra	0.010015	0.019676	0.021509	0.026593	0.732809
Glycine_tabacina	0.009731	0.010165	0.003953	0.036552	0.749677
Lysimachia_arvensis	0.009572	0.012765	0.01898	0.026413	0.766269
Carex_longebrachiata	0.008656	0.018257	0.014226	0.021102	0.781274
Crassula_sieberina	0.008267	0.009811	0.012597	0.025417	0.795605
Panicum_effusum	0.007244	0.010642	0.017172	0.017739	0.808162
Trifolium_sp.	0.007016	0.007799	0.007906	0.02619	0.820323
Facelis_retusa	0.006618	0.008821	0.014565	0.015585	0.831795
Verbena_rigida	0.006415	0.017413	0.021651	0	0.842915
Senecio_madagascariensis	0.005358	0.005665	0.006588	0.019069	0.852203
Wahlenbergia_gracilis	0.005353	0.008923	0.009748	0.015135	0.861483
Desmodium_varians	0.005339	0.006148	0.001318	0.019077	0.870737
Silene_gallica	0.005299	0.006639	0.00812	0.013496	0.879923
Gamochaeta_purpurea	0.004836	0.005746	0.007134	0.01567	0.888307
Sorghum_leiocladum	0.004187	0.020209	0	0.016137	0.895564
Dysphania_pumilio	0.004162	0.00622	0.009211	0.010508	0.902778
Sisyrinchium_rosulatum	0.003094	0.004377	0.003953	0.009769	0.908141
Petrorhagia_nanteuilii	0.002958	0.006355	0	0.009308	0.913269
Angophora_floribunda	0.002942	0.011522	0	0.010197	0.918368
Bidens_pilosa	0.002692	0.00438	0.006362	0.006362	0.923034

Sporobolus_africanus	0.002671	0.006455	0	0.010294	0.927664
Cheilanthes_sieberi	0.002402	0.007397	0.002635	0.008534	0.931828
Vulpia_sp.	0.002395	0.00517	0.001863	0.007991	0.935979
Zornia_dyctiocarpa	0.002387	0.005942	0.009757	0	0.940117
Sporobolus_creber	0.002236	0.004346	0.005816	0.002635	0.943994
Rytidosperma_pilosum	0.002031	0.005407	0	0.006899	0.947515
Euchiton_japonicus	0.001911	0.004195	0.006899	0.001318	0.950827
Wahlenbergia_capillaris	0.00181	0.004285	0.003953	0.001863	0.953964
Cotula_australis	0.001769	0.003776	0.002635	0.003953	0.957031
Dichondra_repens	0.0017	0.003954	0	0.005816	0.959977
Geranium_sp.	0.001597	0.003762	0.001318	0.004499	0.962746
Verbascum_thapsus	0.001572	0.004283	0	0.005582	0.965471
Tricoryne_elatior	0.001486	0.003526	0.001318	0.003953	0.968046
Verbena_bonariensis	0.001393	0.003206	0.00527	0	0.970461
Eragrostis_brownii	0.001343	0.004558	0.00481	0	0.972789
Rumex_brownii	0.0013	0.004617	0	0.005484	0.975042
Gamochaeta_calviceps	0.001295	0.003463	0	0.003953	0.977287
Setaria_parviflora	0.001107	0.003111	0.002635	0.001318	0.979205
Juncus_spp.	0.000997	0.004899	0.002946	0	0.980934
Einadia_nutans	0.000958	0.002686	0.001318	0.002635	0.982594
Sigesbeckia_orientalis	0.00091	0.002527	0.001318	0.002635	0.984172
Digitaria_ramularis	0.000862	0.004153	0	0.004167	0.985666
Tagetes_minuta	0.000816	0.002779	0	0.002635	0.987079
Acaena_novae-zelandiae	0.000799	0.003895	0.002946	0	0.988465
Glycine_clandestina	0.000684	0.002336	0	0.002635	0.98965
Carex_appressa	0.000521	0.002518	0	0.001863	0.990554
Hypericum_gramineum	0.000448	0.00217	0	0.001318	0.99133
Erigeron_sumatrensis	0.000431	0.002117	0.001318	0	0.992078
Lomandra_filiformis	0.000426	0.002062	0	0.001318	0.992817
Sonchus_asper	0.000422	0.002041	0	0.001318	0.993548
Daucus_glochidiatus	0.000411	0.001988	0	0.001318	0.99426
Galium_murale	0.00041	0.001986	0	0.001318	0.994971
Juncus_sp.	0.000385	0.001863	0	0.001318	0.995639
Cerastium_glomeratum	0.000342	0.00165	0	0.001318	0.996232
Dichelachne_micrantha	0.000338	0.001645	0.001318	0	0.996818
Juncus_homalocaulis	0.000338	0.001645	0.001318	0	0.997403
Soliva_sessilis	0.000327	0.001592	0.001318	0	0.997971
Rumex_acetosella	0.000322	0.001567	0.001318	0	0.998529
Hypoxis_hygrometrica	0.000302	0.001467	0.001318	0	0.999053
Modiola_caroliniana	0.000274	0.001318	0	0.001318	0.999528
Acacia_mearnsii	0.000273	0.001313	0	0.001318	1
Anthosachne_scaba	0	0	0	0	1
Briza_minor	0	0	0	0	1
Bromus_hordeaceus	0	0	0	0	1
Carex_breviculmis	0	0	0	0	1
Carex_sp.	0	0	0	0	1

Digitaria_sp.	0	0	0	0	1
Erigeron_sp.	0	0	0	0	1
Euchiton_sphaericus	0	0	0	0	1
Geranium_solanderi	0	0	0	0	1
Hydrocotyle_sp.	0	0	0	0	1
Hypericum_glabra	0	0	0	0	1
Senecio_sp.	0	0	0	0	1
Trifolium_arvense	0	0	0	0	1
Trifolium_repens	0	0	0	0	1
Vulpia_bromoides	0	0	0	0	1

Table S2. Output of SIMPER among KG and ALG+KG sites based on abundance data in spring. Average: mean contribution to the overall dissimilarity, sd: standard deviation of the average dissimilarity, ava: average abundance per plot on ALG+KG sites, avb: average abundance per plot on KG sites, cumsum: ordered cumulative contribution.

species	average	sd	ava	avb	cumsum
Eragrostis_curvula	0.094734	0.081059	0.444385	0.122734	0.162708
Themeda_triandra	0.08909	0.086781	0.576985	0.778713	0.315723
Hypochaeris_radicata	0.028707	0.024869	0.136836	0.093206	0.365028
Plantago_lanceolata	0.017607	0.019785	0.040619	0.074076	0.395269
Poa_labillardierei	0.017272	0.035826	0.037268	0.038911	0.424934
Vulpia_sp.	0.016153	0.019879	0.019283	0.070044	0.452676
Lysimachia_arvensis	0.01545	0.021587	0.057906	0.039674	0.479212
Senecio_madagascariensis	0.015219	0.015195	0.038879	0.046724	0.505351
Paronychia_brasiliana	0.01477	0.020104	0.018276	0.06824	0.530719
Leontodon_saxatilis	0.013393	0.022017	0.03265	0.036927	0.553723
Oxalis_perennans	0.013247	0.016167	0.016131	0.064788	0.576476
Paspalum_dilatatum	0.013193	0.015479	0.047247	0.017297	0.599135
Sorghum_leiocladum	0.01195	0.020205	0.004167	0.043458	0.619658
Carex_longebrachiata	0.011779	0.026669	0.027161	0.028367	0.639888
Glycine_tabacina	0.010697	0.012736	0.001318	0.048212	0.658261
Polycarpon_tetraphyllum	0.010293	0.016928	0.003953	0.052084	0.67594
Carex_inversa	0.010194	0.011721	0.038596	0.019075	0.693449
Facelis_retusa	0.009892	0.008876	0.020024	0.033764	0.710439
Cenchrus_clandestinus	0.009768	0.017211	0.022018	0.029417	0.727215
Microlaena_stipoides	0.009398	0.01305	0.019698	0.028716	0.743356
Verbena_rigida	0.008806	0.018369	0.033061	0.002635	0.758481
Trifolium_sp.	0.00851	0.007636	0.019388	0.042939	0.773097
Silene_gallica	0.008154	0.009428	0.019828	0.026084	0.787102
Crassula_sieberina	0.007456	0.006837	0.014461	0.025099	0.799907
Desmodium_varians	0.007189	0.008354	0.002635	0.030659	0.812254
Gamochaeta_calviceps	0.006994	0.006712	0.011163	0.026425	0.824267

Gamochaeta_purpurea	0.006784	0.007018	0.016357	0.030903	0.835917
Cirsium_vulgare	0.005977	0.011298	0.003953	0.02808	0.846183
Panicum_effusum	0.00516	0.011163	0.015647	0.012263	0.855046
Eragrostis_leptostachya	0.004831	0.011516	0	0.022011	0.863344
Sisyrinchium_rosulatum	0.004824	0.004401	0.014494	0.030625	0.871629
Petrorhagia_nanteuilii	0.004541	0.007686	0.001318	0.019368	0.879428
Erigeron_sp.	0.004383	0.004749	0.007906	0.01866	0.886956
Bothriochloa_macra	0.00393	0.009594	0.00721	0.014427	0.893705
Wahlenbergia_gracilis	0.003811	0.004473	0.007991	0.012404	0.900252
Cheilanthes_sieberi	0.003619	0.009963	0.003953	0.014977	0.906468
Juncus_sp.	0.003571	0.008523	0.01324	0.001863	0.912601
Verbena_bonariensis	0.003271	0.004032	0.009223	0.009223	0.91822
Sporobolus_elongatus	0.003243	0.006446	0.001318	0.013915	0.92379
Euchiton_japonicus	0.003231	0.00673	0.011474	0.004499	0.92934
Briza_minor	0.002539	0.005612	0.009845	0	0.933701
Geranium_sp.	0.002412	0.005608	0.001318	0.008665	0.937843
Zornia_dyctiocarpa	0.002229	0.005096	0.008665	0.001863	0.941672
Verbascum_thapsus	0.002183	0.004266	0.001318	0.008217	0.945421
Euchiton_sphaericus	0.002099	0.003731	0.003953	0.00527	0.949025
Cotula_australis	0.00208	0.004023	0.005044	0.003953	0.952598
Rytidosperma_pilosum	0.002076	0.007397	0	0.008528	0.956162
Wahlenbergia_capillaris	0.001974	0.003672	0.006588	0.001318	0.959553
Dichondra_repens	0.001525	0.003513	0	0.005816	0.962172
Bidens_pilosa	0.001521	0.002883	0.00527	0.002635	0.964784
Angophora_floribunda	0.001505	0.007281	0	0.005893	0.967369
Dichelachne_micrantha	0.001455	0.003425	0.003953	0.001863	0.969867
Rumex_brownii	0.001435	0.003458	0.002635	0.004264	0.972332
Bromus_hordeaceus	0.001262	0.002613	0.001318	0.00527	0.974499
Hypericum_glabra	0.001164	0.005637	0	0.004167	0.976497
Erigeron_sumatrensis	0.001089	0.003847	0.004264	0	0.978367
Tricoryne_elatior	0.001028	0.002852	0.001318	0.002635	0.980133
Galium_murale	0.001022	0.002734	0	0.003953	0.981888
Acaena_novae-zelandiae	0.000902	0.004368	0.004167	0	0.983437
Sporobolus_africanus	0.000703	0.002356	0	0.002635	0.984645
Sonchus_asper	0.000682	0.002356	0.001318	0.001318	0.985816
Geranium_solanderi	0.00065	0.002204	0.002635	0	0.986932
Anthosachne_scaba	0.000577	0.001975	0	0.002635	0.987923
Modiola_caroliniana	0.000519	0.001745	0	0.002635	0.988814
Carex_appressa	0.000476	0.002303	0	0.001863	0.989632
Hypericum_gramineum	0.000474	0.002292	0	0.001863	0.990446
Sporobolus_creber	0.000474	0.002292	0	0.001863	0.99126
Eragrostis_brownii	0.00045	0.002183	0.001863	0	0.992032
Soliva_sessilis	0.000406	0.001969	0.001863	0	0.99273
Trifolium_repens	0.000395	0.001902	0	0.002282	0.993408
Carex_sp.	0.000381	0.001847	0	0.001318	0.994063
Daucus_glochidiatus	0.000359	0.001738	0	0.001318	0.994679

Glycine_clandestina	0.000359	0.001738	0	0.001318	0.995296
Vulpia_bromoides	0.000359	0.001738	0	0.001318	0.995912
Carex_breviculmis	0.000349	0.001689	0	0.001318	0.996512
Trifolium_arvense	0.000345	0.001664	0	0.001863	0.997104
Hydrocotyle_sp.	0.000337	0.001628	0	0.001318	0.997683
Digitaria_sp.	0.000287	0.001392	0.001318	0	0.998176
Cerastium_glomeratum	0.000285	0.001381	0.001318	0	0.998666
Senecio_sp.	0.000285	0.001381	0.001318	0	0.999156
Acacia_mearnsii	0.000246	0.001185	0	0.001318	0.999578
Rumex_acetosella	0.000246	0.001185	0	0.001318	1
Digitaria_ramularis	0	0	0	0	1
Dysphania_pumilio	0	0	0	0	1
Einadia_nutans	0	0	0	0	1
Hypoxis_hygrometrica	0	0	0	0	1
Juncus_homalocaulis	0	0	0	0	1
Juncus_spp.	0	0	0	0	1
Lomandra_filiformis	0	0	0	0	1
Setaria_parviflora	0	0	0	0	1
Sigesbeckia_orientalis	0	0	0	0	1
Tagetes_minuta	0	0	0	0	1

Table S3. Output of SIMPER among KG and ALG+KG sites based on presence / absence data in autumn. Average: mean contribution to the overall dissimilarity, sd: standard deviation of the average dissimilarity, ava: average abundance per plot on ALG+KG sites, avb: average abundance per plot on KG sites, cumsum: ordered cumulative contribution.

species	average	sd	ava	avb	cumsum
Sporobolus_elongatus	0.024097	0.027011	0.291667	0.541667	0.035436
Glycine_tabacina	0.022705	0.022636	0.125	0.583333	0.068825
Senecio_madagascariensis	0.022167	0.026484	0.208333	0.5	0.101423
Trifolium_sp.	0.022042	0.023784	0.25	0.541667	0.133836
Hypochaeris_radicata	0.021746	0.027915	0.583333	0.916667	0.165815
Microlaena_stipoides	0.021273	0.024039	0.5	0.416667	0.197098
Oxalis_perennans	0.020661	0.022017	0.375	0.541667	0.227482
Carex_inversa	0.020594	0.02324	0.416667	0.416667	0.257766
Desmodium_varians	0.020123	0.022372	0.041667	0.5	0.287358
Paspalum_dilatatum	0.019688	0.023577	0.416667	0.25	0.316311
Lysimachia_arvensis	0.019594	0.022197	0.458333	0.333333	0.345125
Plantago_lanceolata	0.019414	0.021366	0.458333	0.375	0.373675
Silene_gallica	0.019085	0.025151	0.166667	0.375	0.40174
Cirsium_vulgare	0.018992	0.024262	0.166667	0.416667	0.429668
Paronychia_brasiliana	0.018916	0.02041	0.375	0.458333	0.457485
Facelis_retusa	0.018495	0.021342	0.166667	0.458333	0.484682
Crassula_sieberina	0.018039	0.020672	0.166667	0.458333	0.511209

Eragrostis_curvula	0.016447	0.025328	0.875	0.708333	0.535395
Gamochaeta_purpurea	0.016383	0.021242	0.208333	0.375	0.559488
Eragrostis_leptostachya	0.014465	0.017719	0.041667	0.416667	0.580759
Sisyrinchium_rosulatum	0.012112	0.017703	0.125	0.291667	0.59857
Panicum_effusum	0.012054	0.016658	0.166667	0.291667	0.616297
Dysphania_pumilio	0.010811	0.015864	0.166667	0.25	0.632195
Poa_labillardierei	0.009686	0.019765	0.125	0.125	0.646438
Sporobolus_creber	0.009625	0.019456	0.166667	0.083333	0.660593
Cenchrus_clandestinus	0.009484	0.017342	0.125	0.166667	0.674539
Wahlenbergia_gracilis	0.009361	0.014359	0.166667	0.208333	0.688305
Themeda_triandra	0.009259	0.019709	0.791667	1	0.701921
Petrorhagia_nanteuilii	0.009158	0.018923	0	0.208333	0.715388
Polycarpon_tetraphyllum	0.008622	0.0164	0.041667	0.208333	0.728067
Wahlenbergia_capillaris	0.008572	0.021618	0.125	0.041667	0.740673
Bidens_pilosa	0.008404	0.013806	0.166667	0.166667	0.753032
Leontodon_saxatilis	0.008404	0.013806	0.166667	0.166667	0.765391
Bothriochloa_macra	0.00788	0.014066	0.125	0.166667	0.776978
Cotula_australis	0.007325	0.015924	0.083333	0.125	0.787751
Carex_longebrachiata	0.007304	0.01592	0.083333	0.125	0.798492
Tricoryne_elatior	0.007201	0.017844	0.041667	0.125	0.809081
Rytidosperma_pilosum	0.006774	0.016183	0	0.166667	0.819043
Dichondra_repens	0.006471	0.015447	0	0.166667	0.828559
Vulpia_sp.	0.006297	0.013139	0.041667	0.166667	0.837819
Verbena_rigida	0.006168	0.017616	0.125	0	0.846889
Verbascum_thapsus	0.006021	0.016848	0	0.125	0.855743
Geranium_sp.	0.005838	0.014084	0.041667	0.125	0.864328
Sporobolus_africanus	0.005732	0.013265	0	0.166667	0.872757
Setaria_parviflora	0.005595	0.016717	0.083333	0.041667	0.880985
Verbena_bonariensis	0.005537	0.012912	0.166667	0	0.889128
Euchiton_japonicus	0.005444	0.011262	0.166667	0.041667	0.897133
Gamochaeta_calviceps	0.005329	0.014554	0	0.125	0.904969
Zornia_dyctiocarpa	0.004487	0.010184	0.166667	0	0.911567
Cheilanthes_sieberi	0.004435	0.010631	0.083333	0.083333	0.918088
Angophora_floribunda	0.004049	0.013963	0	0.083333	0.924043
Einadia_nutans	0.00404	0.011607	0.041667	0.083333	0.929984
Tagetes_minuta	0.00378	0.013561	0	0.083333	0.935542
Sigesbeckia_orientalis	0.003367	0.009348	0.041667	0.083333	0.940494
Eragrostis_brownii	0.003303	0.011428	0.083333	0	0.945352
Rumex_brownii	0.003184	0.010896	0	0.083333	0.950033
Glycine_clandestina	0.003086	0.010712	0	0.083333	0.954572
Erigeron_sumatrensis	0.00235	0.011906	0.041667	0	0.958027
Hypericum_gramineum	0.002254	0.011274	0	0.041667	0.961342
Juncus_spp.	0.002091	0.010474	0.041667	0	0.964417
Sonchus_asper	0.002019	0.010032	0	0.041667	0.967386
Carex_appressa	0.00192	0.009513	0	0.041667	0.970209
Galium_murale	0.00183	0.009048	0	0.041667	0.972901

Acaena_novae-zelandiae	0.001801	0.008918	0.041667	0	0.975549
Lomandra_filiformis	0.001749	0.008629	0	0.041667	0.978121
Daucus_glochidiatus	0.001675	0.008248	0	0.041667	0.980585
Juncus_sp.	0.001545	0.007584	0	0.041667	0.982857
Cerastium_glomeratum	0.001256	0.006122	0	0.041667	0.984703
Modiola_caroliniana	0.001256	0.006122	0	0.041667	0.98655
Sorghum_leiocladum	0.001256	0.006122	0	0.041667	0.988396
Acacia_mearnsii	0.001149	0.005589	0	0.041667	0.990086
Digitaria_ramularis	0.001149	0.005589	0	0.041667	0.991775
Rumex_acetosella	0.001137	0.005527	0.041667	0	0.993448
Soliva_sessilis	0.001137	0.005527	0.041667	0	0.99512
Dichelachne_micrantha	0.001106	0.005373	0.041667	0	0.996747
Hypoxis_hygrometrica	0.001106	0.005373	0.041667	0	0.998373
Juncus_homalocaulis	0.001106	0.005373	0.041667	0	1
Anthosachne_scaba	0	0	0	0	1
Briza_minor	0	0	0	0	1
Bromus_hordeaceus	0	0	0	0	1
Carex_breviculmis	0	0	0	0	1
Carex_sp.	0	0	0	0	1
Digitaria_sp.	0	0	0	0	1
Erigeron_sp.	0	0	0	0	1
Euchiton_sphaericus	0	0	0	0	1
Geranium_solanderi	0	0	0	0	1
Hydrocotyle_sp.	0	0	0	0	1
Hypericum_glabra	0	0	0	0	1
Senecio_sp.	0	0	0	0	1
Trifolium_arvense	0	0	0	0	1
Trifolium_repens	0	0	0	0	1
Vulpia_bromoides	0	0	0	0	1

Table S4. Output of SIMPER among KG and ALG+KG sites based on presence / absence data in spring. Average: mean contribution to the overall dissimilarity, sd: standard deviation of the average dissimilarity, ava: average abundance per plot on ALG+KG sites, avb: average abundance per plot on KG sites, cumsum: ordered cumulative contribution.

species	average	sd	ava	avb	cumsum
Facelis_retusa	0.021303	0.017095	0.208333	0.75	0.034495
Gamochaeta_calviceps	0.018812	0.017401	0.208333	0.625	0.064955
Sisyrinchium_rosulatum	0.018148	0.017804	0.458333	0.916667	0.094342
Crassula_sieberina	0.018078	0.017454	0.208333	0.583333	0.123614
Glycine_tabacina	0.017748	0.016341	0.041667	0.583333	0.152352
Senecio_madagascariensis	0.017684	0.017465	0.375	0.666667	0.180987
Trifolium_sp.	0.016849	0.016983	0.458333	0.875	0.208269
Silene_gallica	0.016523	0.01716	0.375	0.541667	0.235024
Desmodium_varians	0.016234	0.015859	0.083333	0.541667	0.261311
Paronychia_brasiliana	0.016099	0.016255	0.291667	0.541667	0.287379

Gamochaeta_purpurea	0.015928	0.016705	0.5	0.583333	0.31317
Carex_inversa	0.015878	0.016729	0.5	0.416667	0.33888
Oxalis_perennans	0.015826	0.016424	0.458333	0.541667	0.364506
Plantago_lanceolata	0.015684	0.016836	0.583333	0.583333	0.389901
Erigeron_sp.	0.015496	0.016332	0.25	0.5	0.414993
Lysimachia_arvensis	0.015446	0.017077	0.583333	0.666667	0.440004
Polycarpon_tetraphyllum	0.015438	0.016429	0.125	0.5	0.465002
Vulpia_sp.	0.015289	0.015842	0.375	0.5	0.489759
Paspalum_dilatatum	0.014715	0.016547	0.416667	0.291667	0.513585
Microlaena_stipoides	0.013076	0.015563	0.291667	0.333333	0.534758
Verbena_bonariensis	0.012885	0.016105	0.291667	0.291667	0.555622
Wahlenbergia_gracilis	0.011901	0.014479	0.166667	0.375	0.574893
Hypochaeris_radicata	0.010825	0.016009	0.708333	0.916667	0.59242
Cirsium_vulgare	0.01044	0.015085	0.125	0.291667	0.609326
Eragrostis_curvula	0.010381	0.016213	0.958333	0.708333	0.626135
Sorghum_leiocladum	0.010265	0.016096	0.041667	0.291667	0.642757
Petrorhagia_nanteuilii	0.010135	0.015951	0.041667	0.291667	0.659168
Leontodon_saxatilis	0.008363	0.012919	0.208333	0.166667	0.67271
Euchiton_sphaericus	0.00796	0.014251	0.125	0.166667	0.685599
Verbena_rigida	0.007949	0.013952	0.208333	0.083333	0.69847
Cenchrus_clandestinus	0.007852	0.01306	0.166667	0.166667	0.711184
Poa_labillardierei	0.007757	0.01378	0.125	0.166667	0.723745
Wahlenbergia_capillaris	0.007659	0.014384	0.208333	0.041667	0.736146
Verbascum_thapsus	0.007448	0.014126	0.041667	0.208333	0.748206
Themeda_triandra	0.007143	0.014604	0.791667	1	0.759773
Cotula_australis	0.00687	0.013342	0.125	0.125	0.770896
Cheilanthes_sieberi	0.006835	0.012137	0.125	0.166667	0.781964
Euchiton_japonicus	0.00679	0.012072	0.166667	0.125	0.792959
Sporobolus_elongatus	0.006709	0.012562	0.041667	0.208333	0.803822
Briza_minor	0.006557	0.012981	0.208333	0	0.814439
Juncus_sp.	0.005865	0.0123	0.166667	0.041667	0.823935
Eragrostis_leptostachya	0.005837	0.011708	0	0.208333	0.833387
Geranium_sp.	0.005796	0.012037	0.041667	0.166667	0.842771
Panicum_effusum	0.005693	0.010838	0.166667	0.083333	0.851989
Bidens_pilosa	0.005641	0.010716	0.166667	0.083333	0.861124
Carex_longebrachiata	0.0053	0.011268	0.083333	0.125	0.869706
Bromus_hordeaceus	0.005118	0.010655	0.041667	0.166667	0.877992
Dichelachne_micrantha	0.005079	0.012043	0.125	0.041667	0.886216
Dichondra_repens	0.004994	0.011406	0	0.166667	0.894303
Zornia_dyctiocarpa	0.004984	0.010393	0.166667	0.041667	0.902373
Bothriochloa_macra	0.004593	0.009664	0.083333	0.125	0.909811
Rumex_brownii	0.004583	0.011334	0.083333	0.083333	0.917232
Tricoryne_elatior	0.00391	0.010923	0.041667	0.083333	0.923562
Galium_murale	0.00358	0.009633	0	0.125	0.929358
Rytidosperma_pilosum	0.003469	0.009412	0	0.125	0.934975
Sonchus_asper	0.002745	0.009537	0.041667	0.041667	0.939421

Erigeron_sumatrensis	0.002744	0.009284	0.083333	0	0.943864
Geranium_solanderi	0.002621	0.008871	0.083333	0	0.948107
Sporobolus_africanus	0.00251	0.008476	0	0.083333	0.952172
Modiola_caroliniana	0.002352	0.00797	0	0.083333	0.95598
Anthosachne_scaba	0.002175	0.007372	0	0.083333	0.959502
Hypericum_glabra	0.001485	0.007286	0	0.041667	0.961907
Carex_sp.	0.001337	0.006532	0	0.041667	0.964072
Angophora_floribunda	0.001294	0.006316	0	0.041667	0.966167
Carex_appressa	0.001294	0.006316	0	0.041667	0.968262
Hydrocotyle_sp.	0.001294	0.006316	0	0.041667	0.970358
Hypericum_gramineum	0.001294	0.006316	0	0.041667	0.972453
Sporobolus_creber	0.001294	0.006316	0	0.041667	0.974548
Trifolium_arvense	0.001254	0.006114	0	0.041667	0.976579
Carex_breviculmis	0.001216	0.005925	0	0.041667	0.978548
Daucus_glochidiatus	0.001216	0.005925	0	0.041667	0.980517
Glycine_clandestina	0.001216	0.005925	0	0.041667	0.982486
Vulpia_bromoides	0.001216	0.005925	0	0.041667	0.984456
Eragrostis_brownii	0.001213	0.00587	0.041667	0	0.98642
Acaena_novae-zelandiae	0.001178	0.005699	0.041667	0	0.988327
Cerastium_glomeratum	0.001178	0.005699	0.041667	0	0.990235
Senecio_sp.	0.001178	0.005699	0.041667	0	0.992142
Acacia_mearnsii	0.001031	0.005003	0	0.041667	0.993812
Rumex_acetosella	0.001031	0.005003	0	0.041667	0.995481
Trifolium_repens	0.000959	0.004645	0	0.041667	0.997034
Digitaria_sp.	0.000916	0.004418	0.041667	0	0.998517
Soliva_sessilis	0.000916	0.004418	0.041667	0	1
Digitaria_ramularis	0	0	0	0	1
Dysphania_pumilio	0	0	0	0	1
Einadia_nutans	0	0	0	0	1
Hypoxis_hygrometrica	0	0	0	0	1
Juncus_homalocaulis	0	0	0	0	1
Juncus_spp.	0	0	0	0	1
Lomandra_filiformis	0	0	0	0	1
Setaria_parviflora	0	0	0	0	1
Sigesbeckia_orientalis	0	0	0	0	1
Tagetes_minuta	0	0	0	0	1
